# Epidemiological Exploration of *Cryptosporidium* spp., *Giardia intestinalis*, *Enterocytozoon bieneusi*, and *Blastocystis* spp. in Yaks: Investigating Ecological and Zoonotic Dynamics in Lhasa, Xizang

**DOI:** 10.3390/vetsci12050504

**Published:** 2025-05-20

**Authors:** Yaru Ji, Munwar Ali, Chang Xu, Jia Wang, Md. F. Kulyar, Shah Nawaz, Khalid Mehmood, Mingming Liu, Kun Li

**Affiliations:** 1 College of Veterinary Medicine, Nanjing Agricultural University, Nanjing 210095, China; 13653412391@163.com (Y.J.); drmunwarali06@gmail.com (M.A.);; 2 MOE Joint International Research Laboratory of Animal Health and Food Safety, College of Veterinary Medicine, Nanjing Agricultural University, Nanjing 210095, China; 3 College of Veterinary Medicine, Huazhong Agricultural University, Wuhan 430070, China; 4 Department of Parasitology, Faculty of Veterinary and Animal Sciences, The Islamia University of Bahawalpur, Bahawalpur 63100, Pakistan; 5 School of Basic Medicine, Hubei University of Arts and Science, Xiangyang 712000, China

**Keywords:** *Cryptosporidium*, *Giardia*, *Enterocytozoon*, *Blastocystis*, prevalence, yaks

## Abstract

The yak (*Bos grunniens*), prevalent at an altitude between 3000 and 5000 m above sea level, provides the local inhabitants with meat, milk, leather, fuel (dung), and transport. However, intestinal zoonotic parasites seriously endanger its holistic well-being. The prime concern of this study is to investigate the prevalence of four globally ubiquitous zoonotic enteric protozoans, namely *Cryptosporidium* spp., *G. intestinalis*, *Blastocystis* spp., and *E. bieneusi* in yaks from different areas of Lhasa, Xizang. In the given study, 377 yak fecal samples from various regions in Lhasa were obtained, including 161 samples from Linzhou County, 66 samples from Dangxiong County, and 150 samples from the Nimu County cattle farms. Molecular identification of these protozoans was done after amplification using PCR and sequencing of PCR-positive samples, and further phylogenetic analysis was performed. The results indicated that the prevalence of *Cryptosporidium* spp., *G. intestinalis*, *E. bieneusi*, and *Blastocystis* spp. in yak farms in Linzhou County was 48.5, 22.9, 47.8, and 90.7%; 65.2, 13.6, 72.7, and 87.9% in Dangxiong County; and 56.0, 29.3, 58.0, and 80.0%, respectively, in Nimu County. The results of this study provide a basic reference for preventing and controlling intestinal parasites in yaks in Lhasa, Xizang.

## Simple Summary

Protozoan parasites are essential in the livestock industry globally, especially in yaks, because of their grazing-based feeding model. However, the exact prevalence of these protozoan parasites is still unknown. In the given study, 377 yak fecal samples were collected from three regions in Lhasa: Linzhou, Dangxiong, and Nimu County. The prime concerns of this study were to estimate the prevalence of *Cryptosporidium* spp., *Giardia intestinalis* (*G. intestinalis*), *Enterocytozoon bieneusi* (*E. bieneusi*), and *Blastocystis* spp. in the given samples and assess the prevalence of these parasites in these three regions to identify their exact share in the overall global prevalence of these four protozoal parasites. The results demonstrated that the prevalence of *Cryptosporidium* spp., *G. intestinalis*, *E. bieneusi*, and *Blastocystis* spp. in Linzhou County was 48.5, 22.9, 47.8, and 90.7%; 65.2, 13.6, 72.7, and 87.9% in Dangxiong County; and was 56.0, 29.3, 58.0, and 80.0%, respectively, in Nimu County. In conclusion, a significant prevalence of these four parasites was detected in the study areas. After the estimation of the prevalence of these parasites, better preventive and control measures can be implemented.

## 1. Introduction

Parasitic diseases are a major hurdle to animal production [1,2], especially for yaks [3], because of their exposure to diverse environmental conditions. Yaks are mammals of the family Bovidae, mainly distributed in the high-altitude areas of the Qinghai Tibet Plateau and its surroundings above 3000 m in China [4,5]. About 16 million domestic yaks and 15–20 thousand wild yaks are present, of which about 95% are in the rangelands of China, being a first-class protected animal in China among the top endangered species [6,7,8]. Yak is known as the “boat of the plateau”, an important animal for the local population, as its fur can be used in the textile industry to make tents, its hide is used in the leather industry, and its dung is used as fuel for Tibetan herders; yaks produce approximately 147.6–487.2 kg fresh milk per lactation [5], providing the local population with more than 90% of the milk and milk products and 50% of the total meat required [9]. That’s why losses due to parasitic diseases not only affect the bovine industry but also have a significant impact on people’s lives [1,10].

Parasites, such as *Cryptosporidium* spp., *G. intestinalis*, *Blastocystis* spp., and *E. bieneusi*, have been detected in a variety of animals, like cattle, sheep, goats, pigs, alpaca, horses, rabbits, dogs, cats, humans, and birds, with varying prevalence in different regions [11,12,13,14,15]. Transmission occurs via the fecal–oral route through direct contact with infected individuals, contaminated water, or feed/food resources [16,17]. About 42 species of *Cryptosporidium*, 8 assemblages (A–H) of *G. duodenalis* [16,17], 11 major groups of *E. bieneusi* genotypes, and different genotypes of *Blastocystis* based on phylogenetic analyses have been detected in different animals [18,19], necessitating the estimation of their true prevalence.

Cryptosporidiosis is a proto-zoonotic disease and a significant cause of morbidity and mortality in pre-weaned cattle calves (associated estimated cost at £34 per affected calf, excluding labor costs), lambs (weight reduction: about 2.6 kg at slaughter compared to uninfected counterparts), and 1–2-month-old yaks [20,21]. Previous studies have also indicated a prevalence of *Cryptosporidium* spp. in yaks, Tibetan sheep, and camels within the range of 2.0–15.0%, and it has also been detected in various animals in Qinghai [22,23,24]. In Naqu, China, the prevalence of *C. parvum* was found to be 9.1–16.7%, respectively [25].

*Cryptosporidium* spp. have been detected in various animals, like yaks; similarly, *E. bieneusi* is another fungus that has been found in cattle, calves, and yaks [26,27]. Although it is found to be pathogenic in immunocompromised individuals, no definitive pieces of evidence of disease or mortality have been found in bovids. Its prevalence in these hosts highlights its zoonotic potential and ecological significance, being found in soil, water sources, and animal feces [21,22]. *Blastocystis* spp. have been detected in various animals, e.g., from two French zoos [28], Southeast Asian animals [29], and livestock, pets, and zoo animals in Japan [30]. Similarly, *G. intestinalis*, another zoonotic protozoan parasite that primarily infects the small intestine, has been detected in pigs [31,32], dairy cattle [33], and 1–2-month-old highland yaks (as a combined infection with *Cryptosporidium* spp.) [23]. The infection rate of yaks infected with *G. intestinalis* varies, with 6.00% [28], 1.92% [29], 10.4% [30], etc. In addition, the case prevalence of *Blastocystis* spp. up to 44.1% has been reported in pigs [25,34,35], but data regarding yaks is still insufficient.

Our research mainly investigates the prevalence of four protozoan parasites in yaks with three different age groups experiencing different feeding models (e.g., grazing, house-fed) in three distinct regions in Lhasa and Xizang. After molecular epidemiological investigations of these parasites, researchers can better direct future preventive and control strategies.

## 2. Materials and Methods

### 2.1. Collection of Fecal Samples

**Figure 1:**
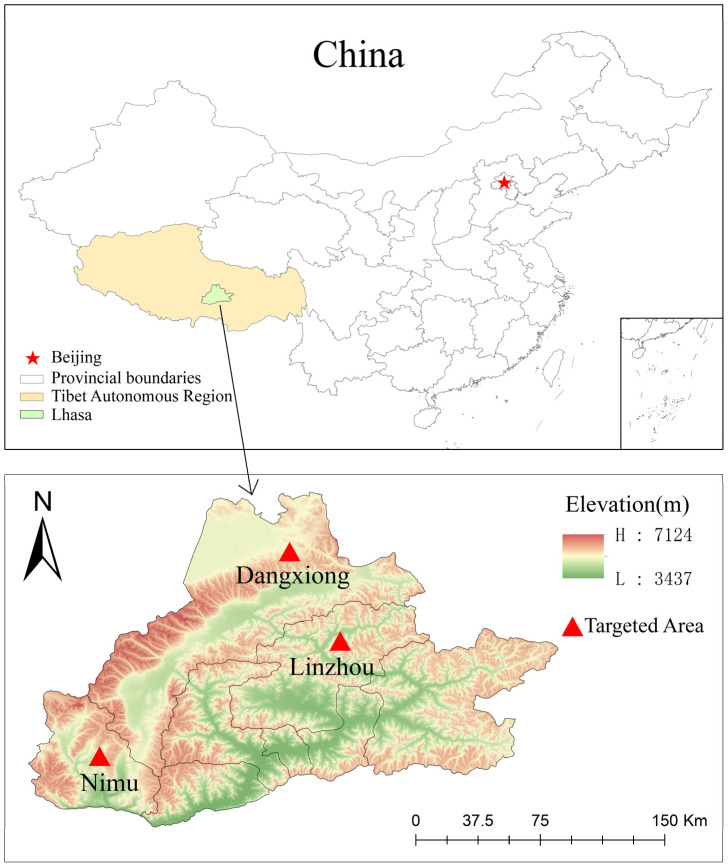
Map showing targeted areas for sample collection. Counties and locations in Lhasa, Xizang, where samples were collected in the given trial. The specific sampling locations were Linzhou, Dangxiong, and Nimu Counties. These three counties are mentioned in the map (GS(2024)0650).

During April 2023, a total of 377 fecal samples were collected from ranches in Linzhou County, Dangxiong County, and Nimu County, Lhasa City, Tibet Autonomous Region, China, at an average altitude of about 4200 m and an average annual temperature of 7.5 °C (Figure 1 and Table 1). The number of yaks in each pasture was different. In Linzhou County, there were 161 yaks, including 40 yak calves, 76 yaks aged 1–2 years, 38 adult yaks, and 7 grazing yaks. There were 66 yaks in Dangxiong County (age and feeding method unknown). There were 150 yaks (age unknown) in Nimu County, including 78 captive yaks and 72 grazing yaks. Yaks were of the same breed and very healthy. No use of antibiotics was recorded, and no intervention was made except for the fecal collection. After collection with sterile cotton swabs, the fresh rectal samples were subsampled (about 5 g) from the middle to avoid contamination with flooring and bedding. After that, the samples were put in frozen storage tubes, rapidly frozen with liquid nitrogen, and transported to the Veterinary Laboratory of Nanjing Agricultural University for further analysis. After careful transportation, the samples were placed at −80 °C in the laboratory to further study the prevalence of four protozoan parasites.

### 2.2. Ethical Statement

The experiments with yaks were conducted after approval from the Animal Welfare Committee on the Ethics of Animal Care and Use, Nanjing Agricultural University (NJAU.No.20230413054).

### 2.3. Total DNA Extraction

All collected fecal samples’ total genomic DNA (gDNA) was extracted using a commercial DNA extraction kit (Item ID: D2100, Solarbio Science & Technology Co., Ltd., Beijing, China). Following the manufacturer’s recommended procedure, genomic DNA was extracted from 0.2 g of feces using a fecal DNA kit and stored at −20 °C before further investigation.

### 2.4. Gene Amplification and DNA Electrophoresis

The detection of parasites’ DNA was performed using PCR technology to amplify the 18S SSU rRNA genes of *G. intestinalis*, *Cryptosporidium* spp., and *Blastocystis* spp., as well as the ITS genes of *E. bieneusi*. Among them, *Cryptosporidium* spp., *G. intestinalis*, and *E. bieneusi* used nested PCR, and *Blastocystis* spp. used endpoint PCR. The primer pairs used in this study were guided by established results [36,37,38,39] (Table 1). The primers Bs-RD5-F/Bs-BhRDr-R are commonly used to detect *Blastocystis* spp. by targeting the SSU rRNA gene.

While these primers have been validated for specificity in various studies, there is a theoretical risk of false positives due to dietary or environmental contaminants. However, negative controls have shown no amplification, suggesting minimal cross-reactivity with host DNA [39,40,41].

The PCR reaction mixture for each amplification reaction consisted of 12.5 μL PCR Mix (which also contains Taq DNA polymerase; 1 unit/25 µL of the reaction mixture, which is essential for amplification, dNTPs, and buffer) Item ID: AG11009, Aikerui Biotechnology Co., Ltd., China, Hunan), 1 μL DNA (50–100 ng/μL), 1.0 μL forward and reverse primers (10 Μm), and 9.5 μL high-pressure distilled water, with a total reaction volume of 25 μL. Among them, CK was tested using 1 μL of high-pressure distilled water as a substitute for template DNA. PCR amplification consisted of 35 PCR cycles, with each cycle having an initial pre-denaturation temperature of 95 °C for 5 min (this was required once at the start of the reaction); the denaturation temperature was 95 °C for 15 s; the primer annealing temperature (Tm) is given in Table 1 and was applied for 15 s; and extended at 72 °C for 45 s. The final extension was at 72 °C for 10 min, and finally, it was stored at 4 °C. Then, all PCR products were detected by 2% agarose gel electrophoresis.

### 2.5. Sequencing Procedure and Phylogenetic Analysis

All PCR-positive samples (confirmed from gel electrophoresis) were sent to Bioengineering Co., Ltd. (Nanjing, China) for bidirectional gene sequencing. Firstly, multiple sequence alignment was performed between the SSU rRNA of yak isolates and the reference gene of the SSU rRNA of the genus *Cryptosporidium* in the NCBI database to identify/download closely matched sequences (≥95% identity). Secondly, multiple sequence alignment was performed with the reference genes of SSU rRNA in the NCBI database for the genus *Blastocystis*. The phylogenetic relationship between yak isolates and reference strains was analyzed using MEGA X (10.1.7) to infer the evolutionary relationships among species regarding the protozoan parasites under study, and the distance was calculated using the adjacency method (NJ). After being guided 1000 times, the stability of the branches in the phylogenetic tree was evaluated. The PCR assays employed in this study were highly sensitive, with a detection limit of 1–10 parasites per reaction, as demonstrated in previous research targeting *Cryptosporidium*, *Giardia*, *Cyclospora*, and *Dientamoeba* species [42]. Such sensitivity ensures reliable detection of protozoan DNA even from 0.2 g of fecal material, showing superior sensitivity compared to microscopy, supporting the robustness of our detection approach. Additionally, the combination of optimized DNA extraction protocols using 0.2 g stool samples [43,44] and sensitive PCR assays mitigates the risk associated with limited fecal sample size, allowing accurate prevalence estimation. 

### 2.6. Statistical Analysis

GraphPad Prism (v8.0.2) software was used to analyze the differences in the prevalence of four parasites among yaks in different places and feeding models (house fed and grazing), with a *p*-value < 0.05 considered statistically significant.

## 3. Results

### 3.1. Infection Status of Yaks Against Four Types of Parasites

**Figure 2:**
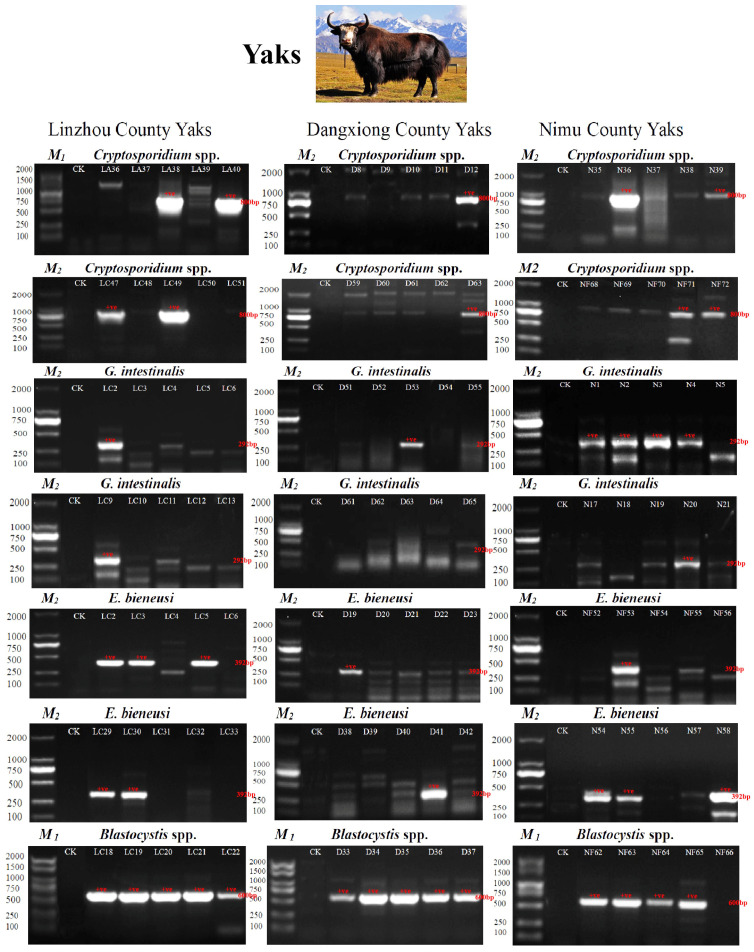
PCR amplification results of SSU rRNA and ITS genes from four protozoan parasites. Two types of Marker ladders were used: M1 and M2. M1: 100, 250, 500, 750, 1000, 1500, 2000 bp. M2: 100, 250, 500, 750, 1000, 2000 bp (M1: Item ID: MD101-01, Vazyme Biotech Co., Ltd., Nanjing, China, M2: Item ID: AG11904, Accurate Biotechnology Co., Ltd., Changsha, China). In Linzhou County, LA indicates yak calves; LC indicates 1–2-year-old yaks. In Dangxiong County, D stands for Dangxiong. In Nimu County, N indicates captive yaks; NF is for grazing yaks. *Cryptosporidium* spp., *G. intestinalis*, and *E. bieneusi* were amplified by nested PCR, and *Blastocystis* spp. amplified by PCR). CK indicates the negative control.

The PCR amplification (positive) results of SSU rRNA and ITS genes of four parasites from three different regions (Linzhou, Dangxiong, and Nimu Counties) are shown in Figure 2 (detailed data are given as Supplementary Materials).

Among 377 yak fecal samples, 161 were from yak farms in Linzhou County, 66 from yak farms in Dangxiong County, and 150 from yak farms in Nimu County. The total prevalence of all samples infected with *Cryptosporidium* spp., *G. intestinalis*, *E. bieneusi*, and *Blastocystis* spp. was found to be 54.4, 23.9, 56.2, and 85.9%, respectively (Table 2).

Out of 161 yak fecal samples from the yak farms in Linzhou County, in total, 78 yaks (48.5%) were found to be positive for *Cryptosporidium* spp., 37 yaks (22.9.0%) tested positive for *G. intestinalis*, 77 yaks (47.8%) were identified as positive for *E. bieneusi*, and 146 yaks (90.7%) tested positive for *Blastocystis* spp. The yaks were further divided into 154 captives and 7 grazing yaks. The captive yaks were further divided into yak calves (40), adult yaks (38), and 1–2-year-old yaks (76). The prevalence of *Cryptosporidium* spp., *G. intestinalis*, *E. bieneusi*, and *Blastocystis* spp. in yak calves was found to be 57.5 (23 specimens), 0.0, 22.5 (9 specimens), and 95.0% (38 specimens), respectively, while in adult yaks, it was found to be 31.6 (12 specimens), 0.0, 26.3 (10 specimens), and 86.8% (33 specimens), respectively. The prevalence in 1–2-year-old yaks was detected to be 56.6 (43 specimens), 47.4 (36 specimens), 67.1 (51 specimens), and 98.7% (75 specimens), while in grazing yaks, it was 0.0, 14.3 (1 specimens), 100.0 (7 specimens), and 0.0% (Table 3).

**Figure 3:**
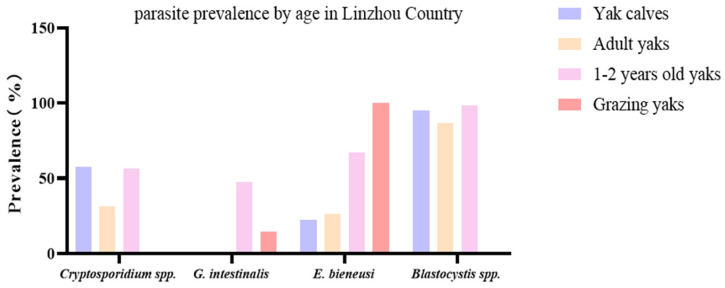
Comparison of the prevalence of protozoan parasites and *E. bieneusi* in yaks from different groups in Linzhou County. This figure compares *G. intestinalis*, *Cryptosporidium* spp., *Blastocystis* spp., and *E. bieneusi* in yak calves, adult yaks, 1–2-year-old yaks, and grazing yaks.

Through fecal testing in Linzhou County, the combined infection of *Cryptosporidium* spp. + *G. intestinalis* + *Blastocystis* spp. + *E. bieneusi* was found to be 17.1% (13 specimens) in 1–2-year-old yaks (Figure 3).

Out of 66 yak fecal samples from the yak farms in Dangxiong County, a total of 43 yaks (65.2%) were identified as positive for *Cryptosporidium* spp., 9 yaks (13.6%) tested positive for *G. intestinalis*, 48 yaks (72.7%) were found to be positive for *E. bieneusi*, and 58 yaks (87.9%) tested positive for *Blastocystis* spp. In Dangxiong County, the prevalence of mixed infection with three types of parasites ranged from 6.1 to 51.5%, and that of the combined prevalence of *Cryptosporidium* spp. + *G. intestinalis* + *Blastocystis* spp. + *E. bieneusi* was 6.1% (4 specimens) (Table 2).

Out of 150 yak fecal samples from Nimu County yak farms, a total of 84 yaks (56.0%) tested positive for *Cryptosporidium* spp., 44 yaks (29.3%) were identified as positive for *G. intestinalis*, 87 yaks (58.0%) were recognized as positive for *E. bieneusi*, and 120 yaks (80.0%) were found to be positive for *Blastocystis* spp. The Nimu County yak farms had 78 captive and 72 grazing yaks. The prevalence of *Cryptosporidium* spp., *G. intestinalis*, *E. bieneusi*, and *Blastocystis* spp. in captive yaks in Nimu County was 53.9 (42 specimens), 55.1 (43 specimens), 48.7 (38 specimens), and 98.7% (77 specimens), respectively; while in grazing yaks in Nimu County, it was 58.3 (42 specimens), 1.4 (1 specimens), 68.1 (49 specimens), and 59.7% (43 specimens), respectively (Table 4).

**Figure 4:**
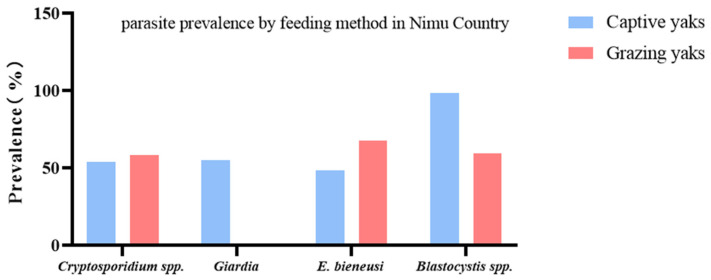
Comparison of the prevalence of protozoan parasites in yaks from different groups in Nimu County. The figure shows the relative prevalence of *G. intestinalis*, *Cryptosporidium* spp., *Blastocystis* spp., and *E. bieneusi* between grazing and captive yaks.

In Nimu County, the prevalence of three species (mixed infection) was 9.3–27.3% (Table 2). On the other hand, the prevalence of combined infection of *Cryptosporidium* spp. + *G. intestinalis* + *Blastocystis* spp. + *E. bieneusi* was detected to be 1.4 (1 specimen) and 16.7% (13 specimens) in captive and grazing yaks, respectively (Table 4, Figure 4).

### 3.2. The Multiregional Difference in Prevalence Among Yaks

Among all samples, the highest prevalence of *Blastocystis* spp. infection was 85.9%; the highest prevalence of dual infection with *E. bieneusi* and *Blastocystis* spp. was 49.6%, while the maximum prevalence of mixed *Cryptosporidium* spp. *+ E. bieneusi + Blastocystis* spp. infections were 8.2% (Table 2).

**Figure 5:**
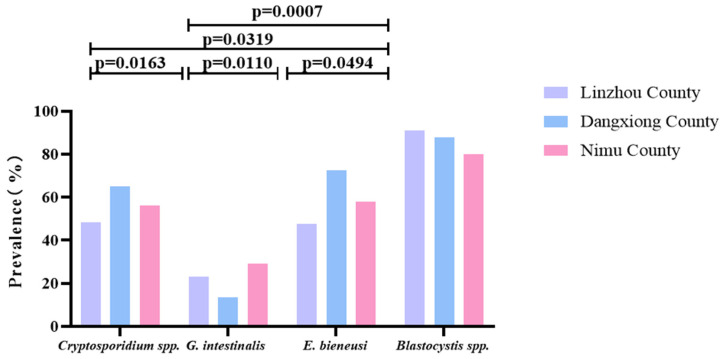
A figure comparing the prevalence of these four parasites in yaks from different regions. The figure shows the relative prevalence of *G. intestinalis*, *Cryptosporidium* spp., *E. bieneusi*, and *Blastocystis* spp. in Linzhou, Dangxiong, and Nimu counties.

As shown in Table 2, among Linzhou, Dangxiong, and Nimu Counties, the prevalence of *Blastocystis* spp. alone was the highest, with a prevalence of 90.7, 87.9, and 80.0%; *G. intestinalis* showed the lowest prevalence of 22.9, 13.6, and 29.3% in Linzhou, Dangxiong, and Nimu Counties, respectively. Among the two types of parasites infected, the combined prevalence of *Cryptosporidium* spp.+* Blastocystis* spp. in Linzhou yaks and Nimu yaks was the highest, with 47.2% and 47.3%, respectively. However, the mixed prevalence of *E. bieneusi + Blastocystis* spp. in Dangxiong County yaks was the highest, with a prevalence of 72.7%. The prevalence of combined infection of *Cryptosporidium* spp. *+ G. intestinalis + E. bieneusi + Blastocystis* spp. was found to be 8.1, 6.1, and 9.3% in Linzhou, Dangxiong, and Nimu Counties, respectively (Table 2). In each region, there was a significant difference between *Cryptosporidium* spp. and *G. intestinalis* (*p* = 0.0163), between *Cryptosporidium* spp. and *E. bieneusi* (*p* = 0.0319), between *G. intestinalis* and *E. bieneusi* (*p* = 0.0110), *G. intestinalis* and *Blastocystis* spp. (*p* = 0.0007), and between *E. bieneusi* and *Blastocystis* spp. (*p* = 0.0494; Figure 5).

### 3.3. Multiple Alignment and Phylogenetic Analysis of Different Gene Sequences

Among all parasite-positive samples, 13 samples were sequenced and stored in GenBank. Among them, one strain of *Cryptosporidium* spp. was isolated from yak calves in Linzhou County (PP439628), and 5 strains of *Cryptosporidium* spp. were isolated from 1–2-year-old yaks in Linzhou County (PP439657, PP439658, PP439660, PP439661, and PP439663). There were 2 strains (PP439445 and PP439446) of *Blastocystis* spp. isolated from grazing yaks in Nimu County, and 5 strains (PP439494, PP439495, PP439497, PP439499, and PP439500) of *Blastocystis* spp. were isolated from 1–2-year-old yaks in Linzhou County.

To accurately identify these isolates and assess their genetic relationships, phylogenetic trees were constructed using MEGA X (version 10.1.7). The resulting tree displays both branching patterns and branch lengths proportional to evolutionary divergence among taxa; the branch lengths are proportional to the number of nucleotide substitutions per site. For *Cryptosporidium* spp., the sequence “HQ149023.1” served as an outgroup to root the tree, providing a reference point for interpreting evolutionary divergence within the ingroup taxa. Similarly, for *Blastocystis* spp., “OR916317.2” served as the outgroup.

**Figure 6:**
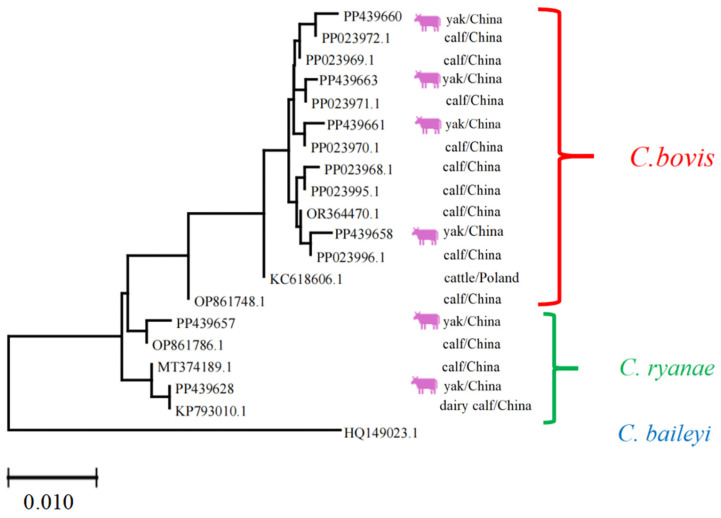
Multiple sequence alignment analysis of the *Cryptosporidium* spp. Lhasa isolates and reference sequences. The purple cattle indicate the sequences acquired from the current study. The scale bar at the bottom (0.010) indicates the genetic distance.

Each sequence was annotated with its GenBank accession number, host species (e.g., yak), and geographic origin (e.g., China). The *Cryptosporidium* phylogeny revealed three distinct clades corresponding to *C. bovis*, *C. ryanae*, and *C. baileyi*. The clustering of sequences from yaks and calves within these clades suggests host-specific adaptations or geographic structuring. The results indicate that regarding *Cryptosporidium* spp., the homology of PP439628, PP439657, PP439660, PP439658, PP439661, and PP439663 strains with previous isolates was 99.0–99.9, 99.6–99.7, 99.6–99.9, 99.6–99.7, and 99.7–99.9%, respectively, confirming the high genetic similarity and validating species identification (Figure 6).

The phylogenetic analysis of *Cryptosporidium* isolates (Figure 6) underscored that sequences clustered distinctly according to *Cryptosporidium* species, as expected. However, no clear clustering was observed based on the geographical origin (Linzhou, Dangxiong, Nimu) or specific animal subgroups. This indicates that the distribution of *Cryptosporidium* species in yaks across the sampled regions is relatively homogeneous, at least for the loci analyzed.

**Figure 7:**
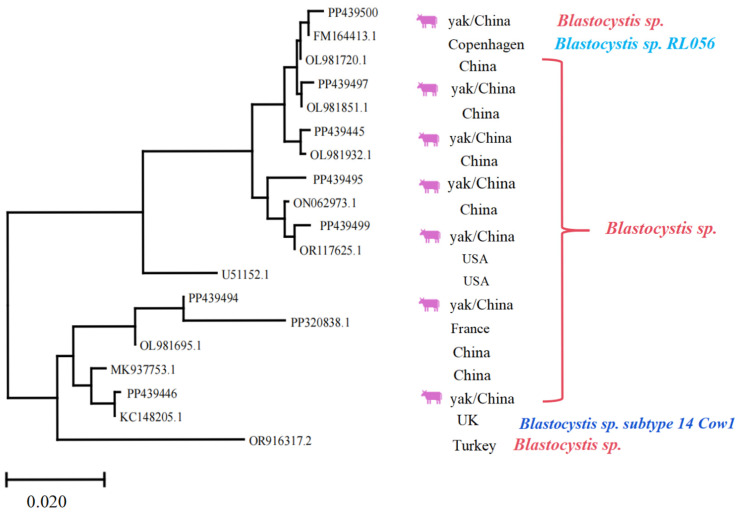
Multiple sequence alignment analysis of the *Blastocystis* spp. Lhasa isolates and reference sequences. The purple cattle indicate the sequences acquired from the current study. The scale bar at the bottom (0.020) indicates the genetic distance.

Regarding *Blastocystis* spp., it was indicated that the homology of PP439445, PP439446, PP439494, PP439495, PP439497, PP439499, and PP439500 with previous isolates was 97.7–99.8, 97.1–99.7, 97.2–98.5, 98.7–99.6, 98.0–99.9, 98.9–99.8, and 98.0–99.7%, respectively. The sequence “OR916317.2” appears to act as an outgroup, rooting the tree and providing a reference point for interpreting evolutionary divergence among the ingroup taxa. The sequences represent diverse geographic origins, including China, the USA, Turkey, France, and the UK, highlighting the widespread distribution and potential for cross-regional transmission (Figure 7). Hence, the phylogenetic tree served a critical role in this study by enabling precise molecular identification of parasite species and subspecies, which is essential for understanding their epidemiology, host specificity, and potential zoonotic risks. Furthermore, the observed genetic diversity and clustering patterns provide valuable insights into the evolutionary relationships and transition dynamics of these protozoan parasites’ population.

## 4. Discussion

Most yaks solely depend upon grazing grasslands throughout the year, generally without any supplementation, and often face a negative energy balance in winter due to less availability of seasonal forages, so a decrease of 25–30% of their body weight, or even more, occurs in winter [45]. That is why sample collection was conducted in April, when the Plateau animals have sufficient food and water, compared to winter (November to March) when these animals undergo starvation and face bitter cold. Hence, the parasitic load can increase, leading to false overestimation of the parasites’ prevalence, e.g., *Cryptosporidium*, *G. intestinalis*, *Blastocystis* spp., and *E. bieneusi* [46].

*Cryptosporidium* spp. can cause zoonotic diseases, leading to severe health and safety issues, and currently, there is no available vaccine [17]. According to previous reports, in dairy cattle, the prevalence in the Gansu region was 4.2% [47], while in calves, it was estimated to be 26.5% in Taiwan [48], and yaks under two years were found with an 18.2% prevalence in the Nagqu region [25]. In the current study, the prevalence of *Cryptosporidium* spp. in three areas ranged from 48.5 to 65.2% (Table 2). Among them, in Linzhou County, captive yaks have a higher prevalence of *Cryptosporidium* spp. than grazing yaks. Differences in water resources, food, environmental factors, animal density, etc., may be the factors for this difference in prevalence. In captive yaks, it was observed that the prevalence of *Cryptosporidium* spp. was higher in yaks under the age of 2 years than in adult yaks (Figure 3), which may be due to the variations in immune response and density of yaks. In another study, the prevalence of *Cryptosporidium* was found to be age-dependent at 49.3% in calves after weaning, 31.7% in 1-year-olds, and 17.4% in mature ones. Overall, 57.1% of yaks were affected with *C. bovis*, 33.7% with *C. range*, 2.0% with *C. andersoni*, 1.0% with *C. ubiquitum*, 1.0% with *C. xiaoi*, 2.0% having a novel genotype, and 3.1% having combined infections of *C. ryanae* and *C. bovis*. During this, a new novel *Cryptosporidium* genotype was detected in the yaks and temporarily named the *Cryptosporidium yak* genotype [49].

In a recent meta-analysis regarding the prevalence of *Cryptosporidium* spp. in yaks, it was found to be 13.5% in northwestern and 4.5% in southwestern China. The prevalence in temporal areas before 2012 was 19.8% and was recorded as more than 6.1% after 2012 [50]. The prevalence of *Cryptosporidium* spp. in winter (20.6%) was higher than in summer (4.8%). Considering age, the yaks aged < 1-year-old had increased prevalence (19.5%) compared to yaks aged ≥ 1 year (16.6%). Additionally, *C. bovis* was found to be highly prevalent. Other factors like geographical conditions (longitude, latitude, temperature, altitude, and precipitation) affect the prevalence of *Cryptosporidium* in yaks [50].

The prevalence of *G. intestinalis* has continuously increased in ruminants and other farm animals [51,52,53] in recent years. The present research estimated the prevalence in the three regions was 22.9, 13.6, and 29.3%, respectively. The prevalence was higher in Nimu County. Similarly, in the Tibetan Plateau Area, Wang et al. [54] have found that the estimated percentage of cases of *G. intestinalis* after investigating 297 samples was 5.0% (15/297) using light microscopic analysis, 6.1% (18/297) using immunofluorescence test (IFT), and was 5.4% (16/297) after performing nested PCR. The average prevalence with the three methods was 5.5% [54].

*Enterocytozoon bieneusi* is also an important zoonotic parasite [39], with prevalence of 47.8, 72.7, and 58.0% in the three regions in the present study. The prevalence in Dangxiong County was significantly higher than that of the other two regions. A similar study was performed by Zhang et al. [55], where seven *E. bieneusi* genotypes were detected in yaks, with five known ones (COS-I, J, BEB4, NESH5, and BEB6) and two novel ones (CHN14 and CHN13), and 7.2% of yaks were positive for this protozoan parasite [55]. *Blastocystis* is another important zoonotic intestinal parasite, with cattle, goats, pigs, deer, sheep, and other animals being its hosts, with prevalence ranging from 14.43 to 100%. Among them, cattle and pigs have a higher prevalence [29]. In the given experiment, the prevalence of *Blastocystis* spp. was high in all three regions, higher than that of *Cryptosporidium* spp., with a prevalence of 90.7, 87.9, and 80.0%, respectively. From Linzhou County and Nimu County, the prevalence of *Blastocystis* spp. in captive yaks was higher than in grazing yaks (Figure 3 and Figure 4), possibly due to different environmental hygiene, dietary conditions, and other factors. In addition, this study found that the prevalence of these four protozoan parasitic infections among infected yaks from different regions differed significantly (Table 2, Figure 5). The difference in prevalence compared to previous studies may be due to other geographical locations, climatic environments, animal density, sample size, and feeding methods [25]. After infection in yaks, pathogens are excreted through the fecal–oral route, and contaminated water and food are transmitted to other livestock or herders [27,56].

In the present study, phylogenetic testing indicated that *Cryptosporidium* isolates from yaks clustered according to species, in alignment with the established results [57]. However, in the current research, no distinct clustering by geographical locations or animal subgroups was detected. This absence of geographic or host-associated clustering may point out a widespread distribution of *Cryptosporidium* spp. among yak populations in Lhasa, possibly as a result of animal movement, common water sources, or other external factors, facilitating gene flow [58,59]. Alternatively, the genetic markers used (e.g., 18S rRNA) may provide insufficient details to detect fine-scale population structure [58]. Similar results have been reported in other studies of *Cryptosporidium* spp. in animals, where high genetic similarity was observed across wide geographical locations [57,59]. Future studies with higher-resolution markers or whole-genome sequencing can lead to the detection of subtle epidemiological patterns.

Yaks can withstand temperature extremes and low oxygen tension because they evolved after intense natural selection regarding their morphology, physiology, and metabolic needs. However, the illness of yaks affects the quality of human life and causes economic losses to the animal husbandry industry [9]. That is why parasitic infections are one of the global issues in both public health and livestock sectors, especially in remote and resource-scarce areas where infections are most likely to occur [49]. So, the current study investigated the prevalence of zoonotic parasites in remote areas. Monitoring the prevalence of infectious pathogens in livestock regarding specific regions, climates, feeding models, and age groups of yaks is very important to formulate effective prevention and control strategies. These strategies include (1) enhanced sanitation protocols for captive yaks to disrupt fecal-oral transmission cycles, (2) rotational grazing systems to reduce environmental contamination in pastures, (3) targeted monitoring of 1–2-year-old yaks, which showed heightened susceptibility to *Cryptosporidium* spp. and *E. bieneusi*, and (4) judicious use of antiparasitics, such as nitazoxanide (for cryptosporidiosis) and albendazole (for giardiasis), although efficacy remains partial and resistance risks necessitate genotype-guided dosing.

This study’s limitations include the lack of zoonotic potential evaluation and *Cryptosporidium* genotyping, hindering public health risk assessment. While the primers used for *Blastocystis* detection are generally specific, the potential for dietary or environmental contamination and the low sequencing rate introduce uncertainty and limit subtype resolution. Future studies should include a positive control and should prioritize sequencing all PCR-positive samples to assess genetic diversity and confirm host specificity, along with prioritizing genotyping, longitudinal studies on seasonal parasite fluctuations, One Health approaches, and tailored antiparasitic treatments for yaks in high-altitude regions, expanding investigations beyond Lhasa.

## 5. Conclusions

This study reports the epidemiological distribution of four important zoonotic parasites in yaks in a particular environment. The results estimated the average prevalence of *Cryptosporidium* spp. (56.5%), *Giardia intestinalis* (22.0%), *E. bieneusi* (59.5%), and *Blastocystis* spp. (86.2%) in three different regions, providing a reference for prevention strategies for these four zoonotic protozoan parasites in specific environmental conditions to reduce their prevalence.

## Figures and Tables

**Table 1:** Primer pairs used in the given experiment.

Gene	Primer	Sequence (5′-3′)	Annealing Temperature (°C)	Fragment Length (bp)	Reference
*SSU* rRNA	CP-SSU-F1	CCCATTTCCTTCGAAACAGGA	55	830	[36]
	CP-SSU-R1	TTCTAGAGCTAATACATGCG			
	CP-SSU-F2	AAGGAGTAAGGAACAACCTCCA	58	800	
	CP-SSU-R2	GGAAGGGTTGTATTATTAGATAAAG			
*SSU* rRNA	GD-Gia2029-F1	AAGTGTGGTGCAGACGGACTC	55	497	[37]
	GD-Gia2150c-R1	CTGCTGCCGTCCTTGGATGT			
	GD-RH11-F1	CATCCGGTCGATCCTGCC	59	292	
	GD-RH4-R2	AGTCGAACCCTGA TTCTCCGCCAGG			
*ITS*	AL4037-F1	GATGGTCATAGGGATG AAGAGCTT	55	410	[38]
	AL4039-R1	AATACAGGATCACTTGGATCCGT			
	AL4038-F2	AGGGATGAAGAGCTTCGGCTCTG	55	392	
	AL4040-R2	AATATCCCTAATACAGGATCACT			
*SSU* rRNA	Bs-RD5-F	ATCTGGTTGATCCTGCCAGT	59	600	[39]
	Bs-BhRDr-R	GAGCTTTTTAACTGCAACAACG			

F: forward primer, R: reverse primer.

**Table 2:** Individual and cumulative prevalence of protozoan parasites and *E. bieneusi* in yaks in Linzhou, Dangxiong, and Nimu Counties.

No. of Positive Samples (*n*)	Linzhou (161)	Dangxiong (66)	Nimu (150)	Total (377)
Parasite Genus/Species	No. Tested/Prevalence% (95% CI Values)			
*Cryptosporidium* spp.	78/48.5 ^a^ (40.7–56.2)	43/65.2 (53.6–76.7)	84/56.0 (48.1–63.9)	205/54.4 (49.4–59.4)
*G. intestinalis*	37/22.9 (16.5–29.5)	9/13.6 (5.4–21.9)	44/29.3 (22.0–36.6)	90/23.9 (19.6–28.2)
*E. bieneusi*	77/47.8 (40.1–55.6)	48/72.7 ^b^ (62.0–83.5)	87/58.0 ^c^ (50.1–65.9)	212/56.2 (51.2–61.2)
*Blastocystis* spp.	146/90.7 ^d^ (86.2–95.2)	58/87.9 ^e^ (80.0–95.8)	120/80.0 (73.6–86.4)	324/85.9 (82.4–89.5)
*Cryptosporidium* spp. *+ G. intestinalis*	20/12.4 (7.3–17.5)	4/6.1 (0.0–11.8)	22/14.7 (9.0–20.3)	46/12.2 (8.9–15.5)
*Cryptosporidium* spp. *+ E. bieneusi*	39/24.2 (17.6–30.8)	35/53.0 (36.5–69.5)	51/34.0 (26.4–41.6)	125/33.2 (24.9–41.5)
*Cryptosporidium* spp. *+ Blastocystis* spp.	76/47.2 (39.5–54.9)	40/60.6 (48.8–72.4)	71/47.3 (39.3–55.3)	187/49.6 (44.6–54.7)
*G. intestinalis + E. bieneusi*	25/15.5 (9.9–21.1)	9/13.7 (5.4–21.9)	21/14.0 (8.5–19.6)	55/14.6 (11.0–18.2)
*G. intestinalis + Blastocystis* spp.	35/21.7 (15.4–28.1)	9/13.7 (5.4–21.9)	44/29.3 (22.0–36.6)	88/23.4 (19.1–27.6)
*E. bieneusi + Blastocystis* spp.	70/43.5 (35.8–51.1)	48/72.7 (62.0–83.5)	66/44.0 (36.1–51.9)	184/48.8 (41.6–56.0)
*Cryptosporidium* spp. *+ G. intestinalis + E. bieneusi*	13/8.1 (3.9–12.3)	4/6.1 (0.0–11.8)	14/9.3 (4.7–14.0)	31/8.2 (5.5–11.0)
*Cryptosporidium* spp. *+ G. intestinalis Blastocystis* spp.	20/12.4 (7.3–17.5)	4/6.1 (0.0–11.8)	22/14.7 (9.0–20.3)	46/12.2 (8.9–15.5)
*Cryptosporidium* spp. *+ E. bieneusi + Blastocystis* spp.	39/24.2 (17.6–30.8)	34/51.5 (34.7–68.3)	41/27.3 (20.2–34.5)	114/30.2 (21.8–38.6)
*G. intestinalis + E. bieneusi + Blastocystis* spp.	24/14.9 (9.4–20.4)	9/13.7 (5.4–21.9)	21/14.0 (8.5–19.6)	54/14.3 (10.8–17.9)
*Cryptosporidium* spp. *+ G. intestinalis + E. bieneusi+ Blastocystis* spp.	13/8.1 (3.9–12.3)	4/6.1 (0.0–11.8)	14/9.3 (4.7–14.0)	31/8.2 (5.5–11.0)

^a^ A significant difference was found in three regions regarding the prevalence of *Cryptosporidium* spp. and *G. intestinalis* (*p* = 0.0163). ^b^ A significant difference was found in the prevalence of *Cryptosporidium* spp. and *E. bieneusi* (*p* = 0.0319). ^c^ The difference was significant in the prevalence of *E. bieneusi* and *G. intestinalis* (*p* = 0.0110). ^d^ The difference was significant in the prevalence of *Blastocystis* spp. and *G. intestinalis* (*p* = 0.0007). ^e^ The difference was significant in the prevalence of *Blastocystis* spp. and *E. bieneusi* (*p* = 0.0494) in the three regions under study. Calculation for 95% confidence intervals (CIs) used the Wald method: $CI=\frac{p\pm 1.96\times \sqrt{p\left(1-p\right)}}{n},\;$ for consistency. In this equation, *p* is the sample proportion, *n* is the sample size, and 1.96 is the Z-score corresponding to a 95% confidence level.

**Table 3:** Prevalence of protozoan parasites and *E. bieneusi* in different groups of yaks in Linzhou County.

	Linzhou County Yaks							
	Captive Yaks						Grazing Yaks	
	Yaks’ Calves		Adults Yaks		1–2 Years Old Yaks			
Protozoan Parasites	No. Tested/ No. Positive	Prevalence (%)	No. Tested/ No. Positive	Prevalence (%)	No. Tested/ No. Positive	Prevalence (%)	No. Tested/ No. Positive	Prevalence (%)
*Cryptosporidium* spp.	23/40	57.5	12/38	31.6	43/76	56.6	0	0.0
*G. intestinalis*	0	0.0	0	0.0	36/76	47.4	1/7	14.3
*E. bieneusi*	9/40	22.5	10/38	26.3	51/76	67.1	7/7	100.0
*Blastocystis* spp.	38/40	95.0	33/38	86.8	75/76	98.7	0	0.0
*Cryptosporidium* spp. *+ G. intestinalis*	0	0.0	0	0.0	20/76 ^a^	26.3	0	0.0
*Cryptosporidium* spp. *+ E. bieneusi*	8/40	20.0	2/38	5.3	29/76	38.2	0	0.0
*Cryptosporidium* spp. *+ Blastocystis* spp.	22/40	55.0	11/38	29.0	43/76	56.6	0	0.0
*G. intestinalis + E. bieneusi*	0	0.0	0	0.0	24/76 ^b^	31.6	1/7	14.3
*G. intestinalis + Blastocystis* spp.	0	0.0	0	0.0	35/76 ^b^	46.1	0	0.0
*E. bieneusi + Blastocystis* spp.	9/40	22.5	10/38	26.3	51/76	67.1	0	0.0
*Cryptosporidium* spp. *+ G. intestinalis + E. bieneusi*	0	0.0	0	0.0	13/76	17.1	0	0.0
*Cryptosporidium* spp. *+ G. intestinalis + Blastocystis* spp.	0	0.0	0	0.0	20/76 ^c^	26.3	0	0.0
*Cryptosporidium* spp. *+ E. bieneusi + Blastocystis* spp.	8/40	20.0	2/38	5.3	29/76	38.2	0	0.0
*G. intestinalis + E. bieneusi + Blastocystis* spp.	0	0.0	0	0.0	24/76 ^d^	31.6	0	0.0
*Cryptosporidium* spp. *+ G. intestinalis + E. bieneusi + Blastocystis* spp.	0	0.0	0	0.0	13/76	17.1	0	0.0

^a^ A significant difference was found in three regions regarding the prevalence of *Cryptosporidium* spp. and *G. intestinalis* (*p* = 0.0163). ^b^ A significant difference was found in the prevalence of *Cryptosporidium* spp. and *E. bieneusi* (*p* = 0.0319). ^c^ The difference was significant in the prevalence of *E. bieneusi* and *G. intestinalis* (*p* = 0.0110). ^d^ The difference was significant in the prevalence of *Blastocystis* spp. and *G. intestinalis* (*p* = 0.0007).

**Table 4:** Prevalence of protozoan parasites and *E. bieneusi* in different groups of yaks in Nimu County.

	Nimu County Yaks			
	Captive Yaks		Grazing Yaks	
Parasites	No. Positive/No. Tested	Prevalence (%)	No. Positive/No. Tested	Prevalence (%)
*Cryptosporidium* spp.	42/78	53.9	42/72	58.3
*G. intestinalis*	43/78	55.1	1/72	1.4
*E. bieneusi*	38/78	48.7	49/72	68.1
*Blastocystis* spp.	77/78	98.7	43/72	59.7
*Cryptosporidium* spp. *+ G. intestinalis*	21/78	26.9	1/72	1.4
*Cryptosporidium* spp. *+ E. bieneusi*	22/78	28.2	29/72	40.3
*Cryptosporidium* spp. *+ Blastocystis* spp.	42/78	53.9	29/72	40.3
*G. intestinalis + E. bieneusi*	20/78	25.6	1/72	1.4
*G. intestinalis + Blastocystis* spp.	43/78	55.1	1/72	1.4
*E. bieneusi + Blastocystis* spp.	38/78	48.7	28/72	38.9
*Cryptosporidium* spp. *+ G. intestinalis + E. bieneusi*	13/78	16.7	1/72	1.4
*Cryptosporidium* spp. *+ G. intestinalis + Blastocystis* spp.	21/78	26.9	1/72	1.4
*Cryptosporidium* spp. *+ E. bieneusi + Blastocystis* spp.	22/78	28.2	19/72	26.4
*G. intestinalis + E. bieneusi +Blastocystis* spp.	20/78	25.6	1/72	1.4
*Cryptosporidium* spp. *+ G. intestinalis + E. bieneusi + Blastocystis* spp.	13/78	16.7	1/72	1.4

## Data Availability

All data are contained within the article/Supplementary Materials.

## Supplementary Materials

The following supporting information can be downloaded at: https://www.mdpi.com/article/10.3390/vetsci12050504/s1, Supplementary Materials: Gel Picture.
