# Supplementary material for: Epidemiological Exploration of Cryptosporidium spp., Giardia intestinalis, Enterocytozoon bieneusi, and Blastocystis spp. in Yaks: Investigating Ecological and Zoonotic Dynamics in Lhasa, Xizang
Source: Vet Sci. 2025 May 20;12(5):504. doi: 10.3390/vetsci12050504 (PMC12115546; doi:10.3390/vetsci12050504)
Supplement: Supplementary file 1 [file vetsci-12-00504-s001.zip › vetsci-3555693-supplementary.pdf]

Gel picture

Linzhou County

1. *Cryptosporidium* spp.

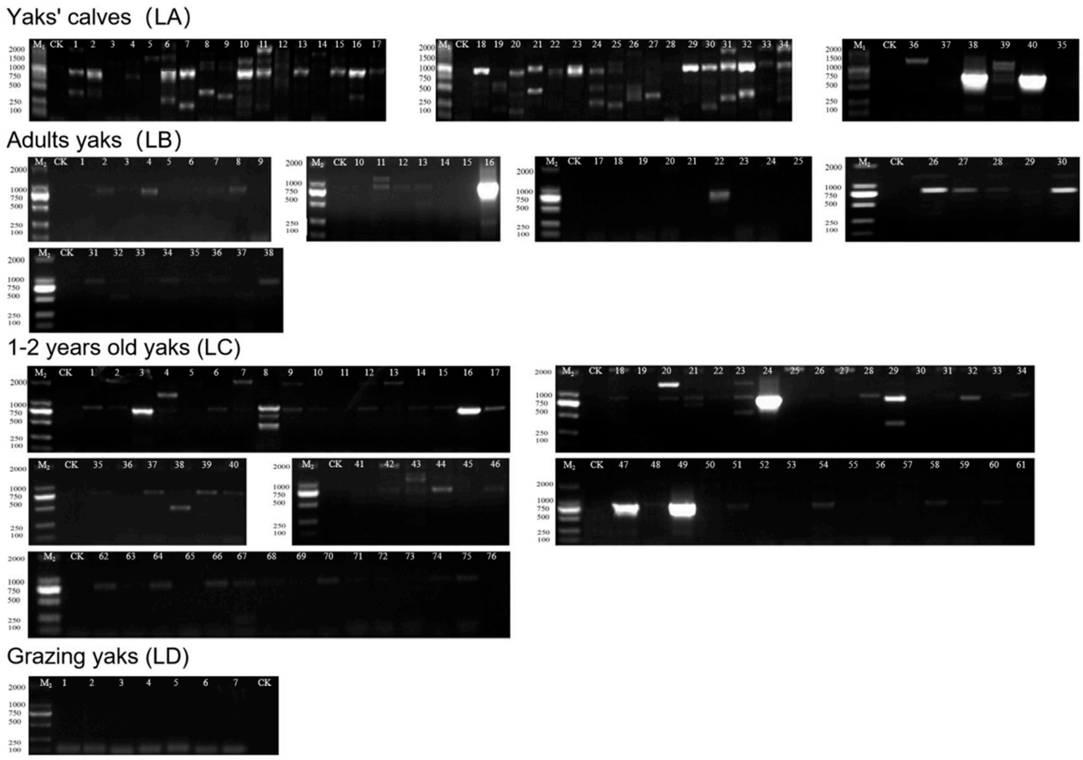

2. *Giardia intestinalis*

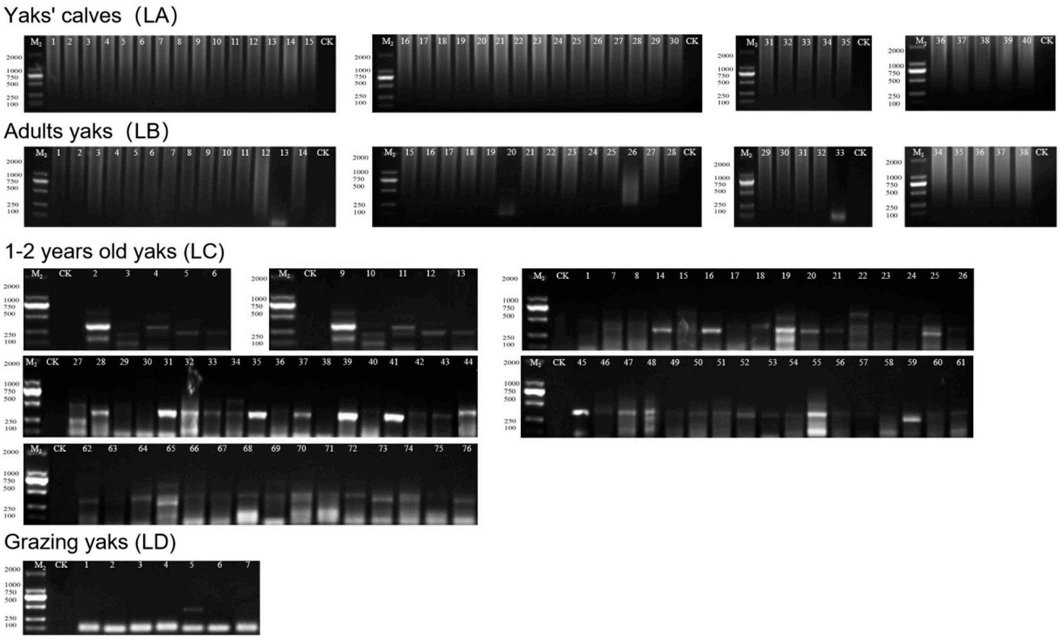

3. *E. bieneusi*

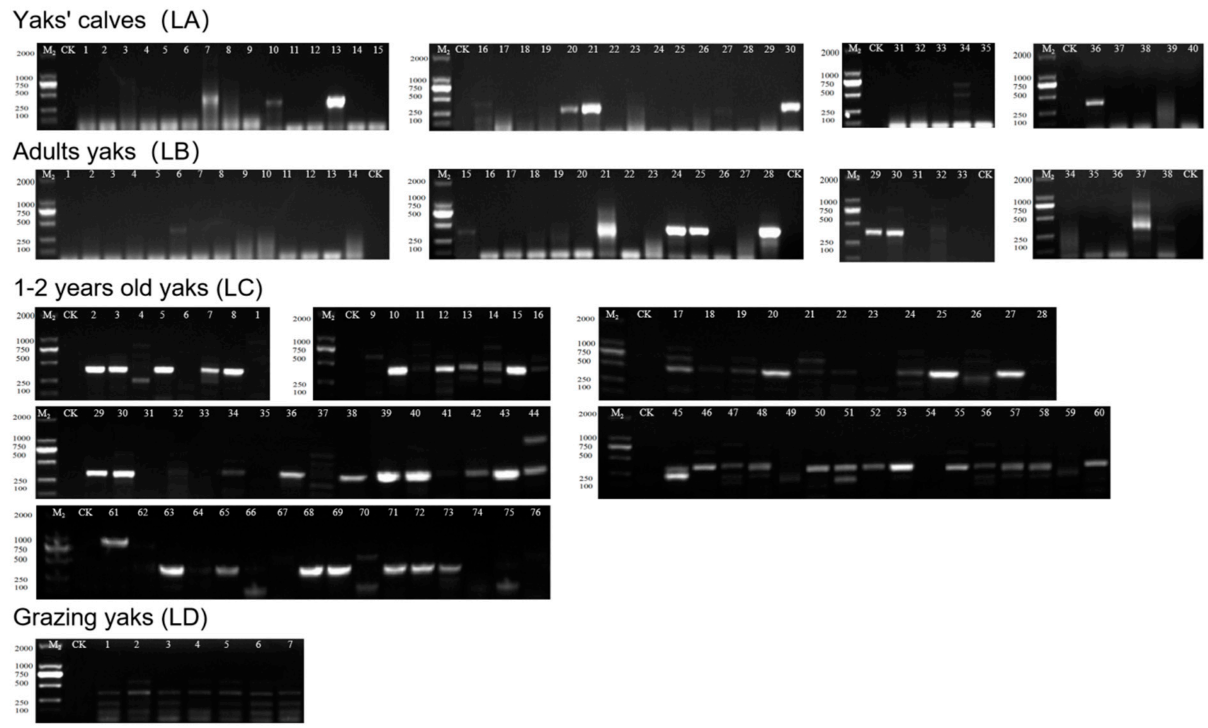

4. *Blastocystis* spp.

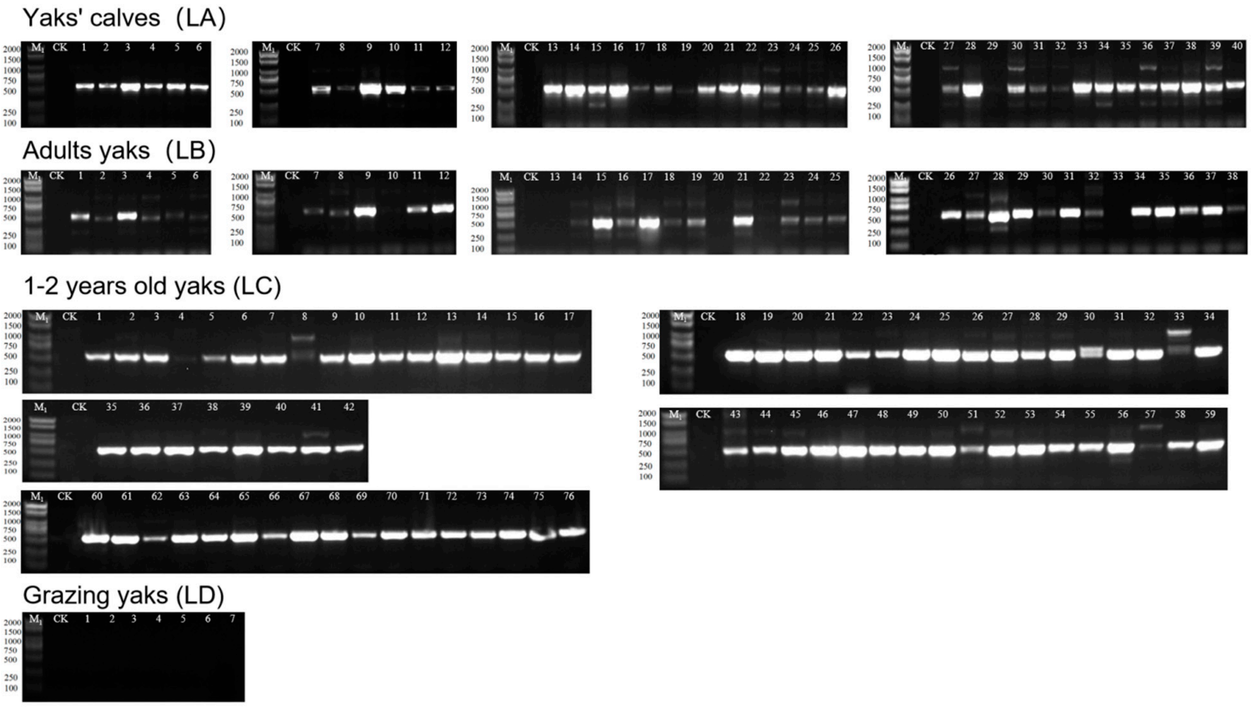

Dangxiong County

Cryptosporidium spp.

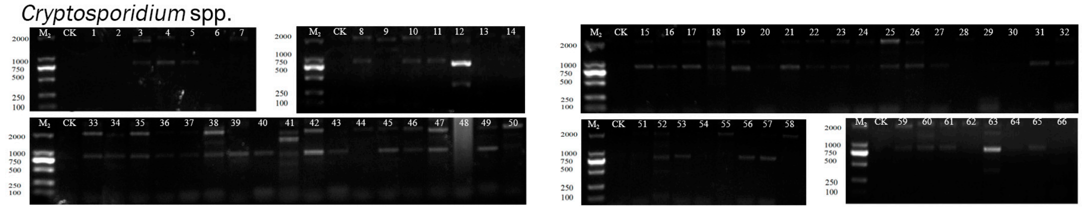

Giardia intestinalis

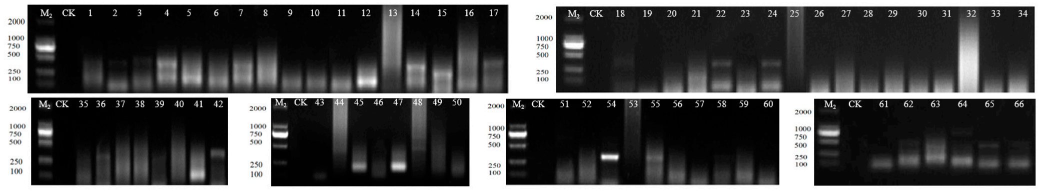

E. bieneusi

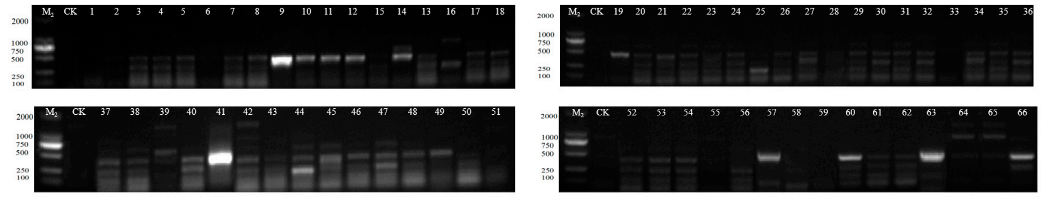

Blastocystis spp.

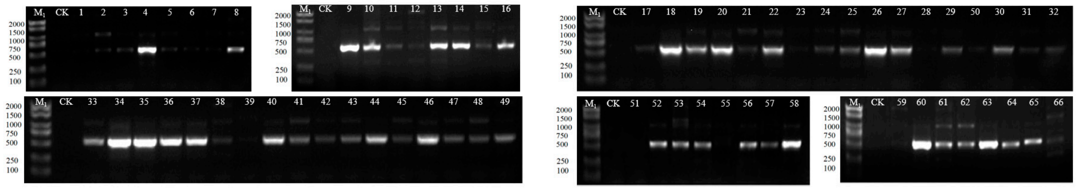

Nimu County

1. *Cryptosporidium* spp.

Captive yaks（N）

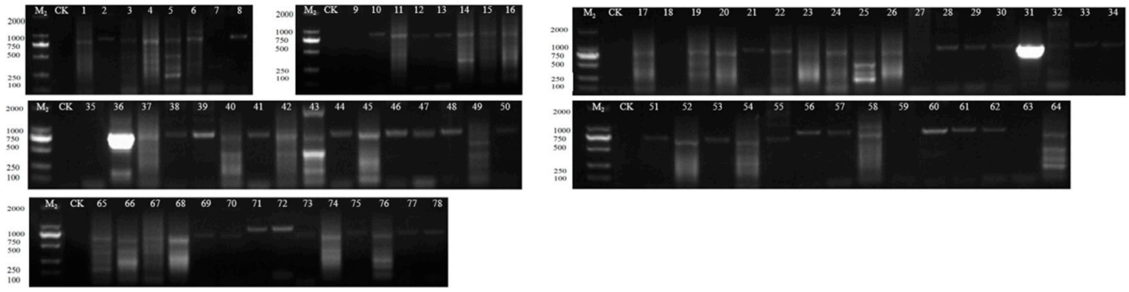

Grazing yaks (NF)

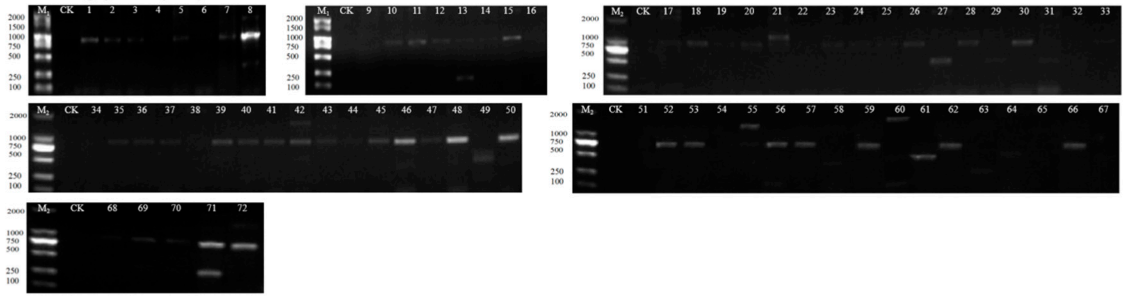

2. *Giardia intestinalis*

Captive yaks（N）

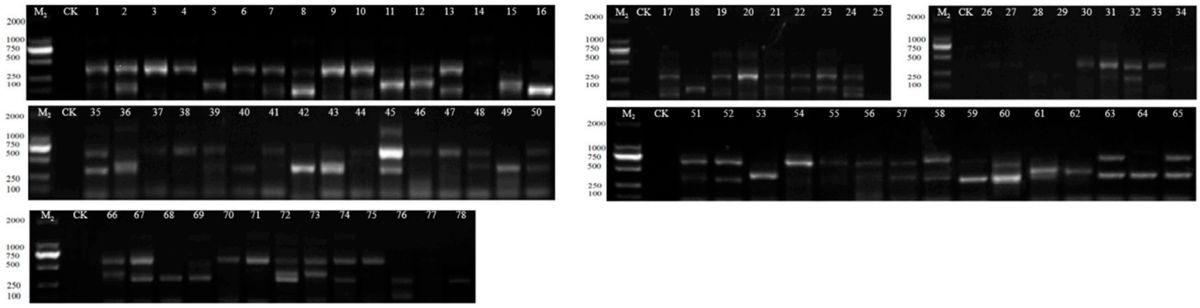

Grazing yaks (NF)

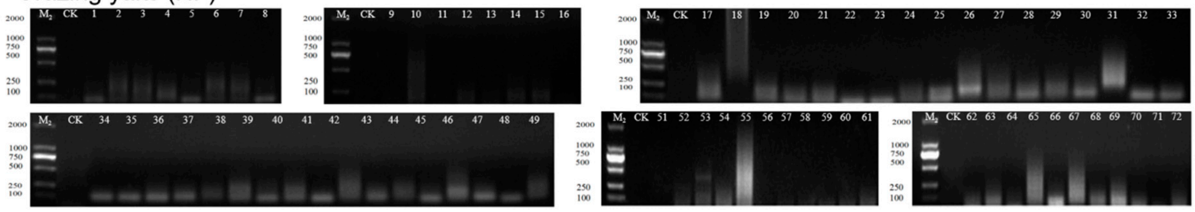

3. *E. bieneusi*

Captive yaks (N)

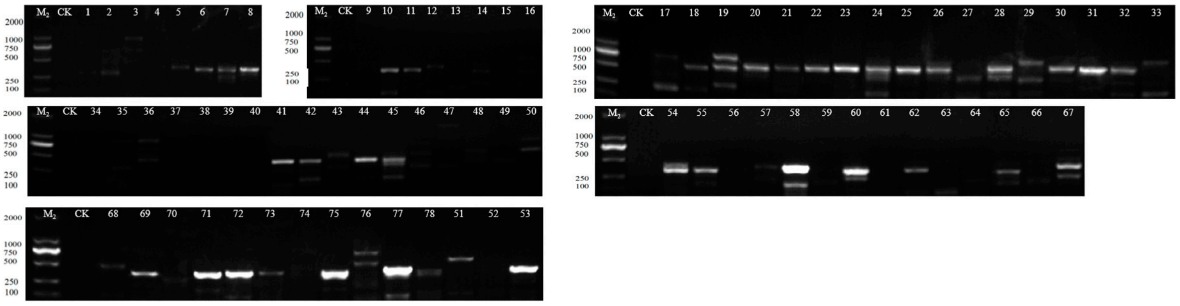

Grazing yaks (NF)

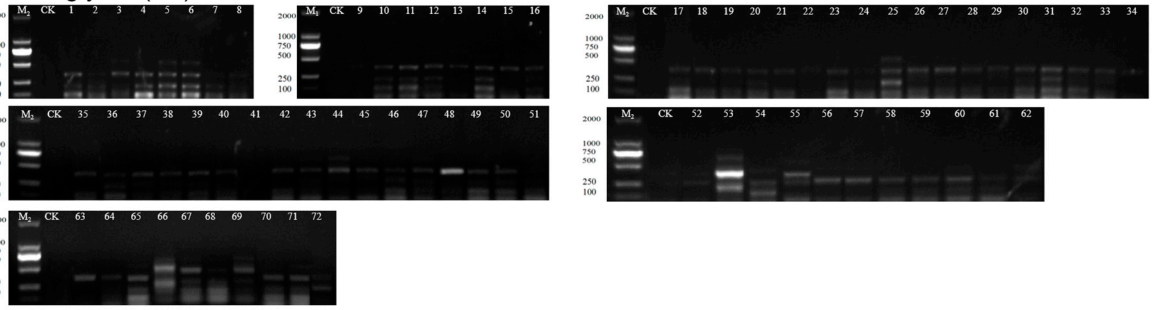

4. *Blastocystis* spp.

Captive yaks (N)

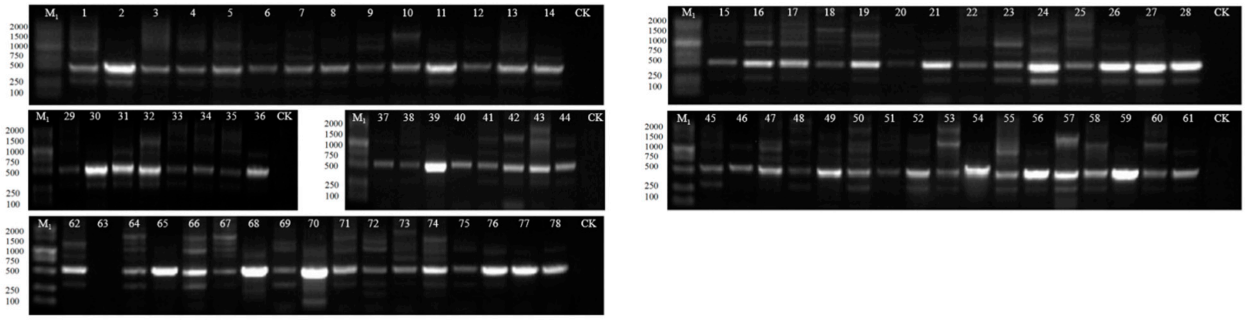

Grazing yaks (NF)

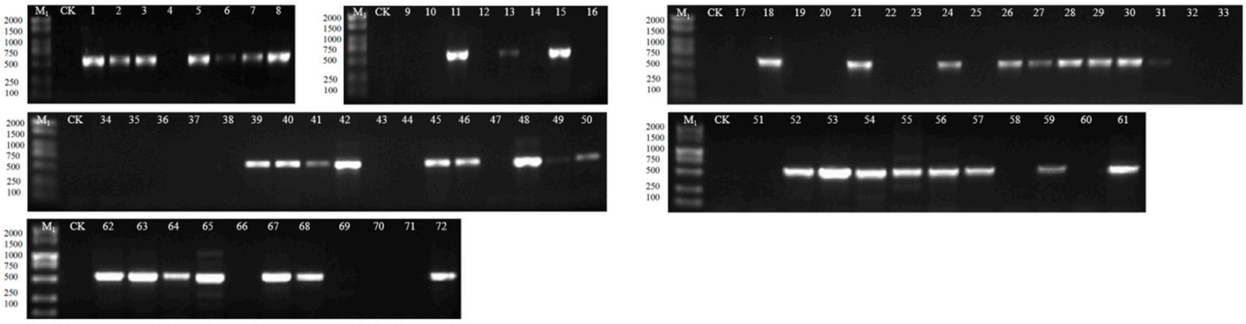

Single parasite infection

Positive samples in Linzhou County	
Parasite	Sample ID
Cryptosporidium spp.	Yak calves（LA）<br>1, 2, 4, 6, 7, 10, 11, 13, 15, 16, 17, 18, 20, 22, 23, 24, 29, 30, 31, 32, 34, 38, 40
	Adult yaks（LB）<br>2, 4, 8, 11, 12, 13, 16, 22, 26, 27, 28, 30
	1–2-year-old yaks (LC)<br>1, 2, 3, 6, 8, 9, 10, 12, 13, 14, 15, 16, 17, 18, 20, 21, 22, 23, 24, 28, 29, 31, 32, 34, 35, 37, 38, 39, 40, 42, 43, 44, 46, 47, 49, 51, 54, 58, 60, 62, 64, 66, 67
	Grazing yaks (LD)<br>NO
Giardia intestinalis	Yak calves（LA）<br>NO
	Adult yaks（LB）<br>NO
	1–2-year-old yaks (LC)<br>2, 4, 9, 11, 14, 16, 18, 19, 20, 21, 22, 25, 28, 31, 32, 33, 34, 35, 37, 39, 41, 43, 44, 45, 47, 48, 50, 51, 52, 53, 55, 56, 59, 61, 62, 65
	Grazing yaks (LD)<br>5
E. bieneusi	Yak calves（LA）<br>7, 10, 13, 16, 20, 21, 30, 34, 36
	Adult yaks（LB）<br>6, 15, 21, 24, 25, 28, 29, 30, 37, 38
	1–2-year-old yaks (LC)<br>2, 3, 5, 7, 8, 10, 11, 12, 13, 14, 15, 16, 17, 18, 19, 20, 21, 22, 24, 25, 27, 29, 30, 32, 34, 36, 38, 39, 40, 42, 43, 44, 46, 47, 48, 50, 51, 52, 53, 55, 56, 57, 58, 60, 63, 65, 68, 69, 71, 72, 73
	Grazing yaks (LD)<br>1, 2, 3, 4, 5, 6, 7
Blastocystis spp.	Yak calves（LA）<br>1, 2, 3, 4, 5, 6, 7, 8, 9, 10, 11, 12, 13, 14, 15, 16, 17, 18, 20, 21, 22, 23, 24, 25, 26, 27, 28, 30, 31, 32, 33, 34, 35, 36, 37, 38, 39, 40
	Adult yaks（LB）<br>1, 2, 3, 4, 5, 6, 7, 8, 9, 11, 12, 14, 15, 16, 17, 18, 19, 21, 23, 24, 25, 26, 27, 28, 29, 30, 31, 32, 34, 35, 36, 37, 38
	1–2-year-old yaks (LC)<br>1, 2, 3, 5, 6, 7, 8, 9, 10, 11, 12, 13, 14, 15, 16, 17, 18, 19, 20, 21, 22, 23, 24, 25, 26, 27, 28, 29, 30, 31, 33, 32, 34, 35, 36, 37, 38, 39, 40, 41, 42, 43, 44, 45, 46, 47, 48, 49, 50, 51, 52, 53, 54, 55, 56, 57, 58, 59, 60, 61, 62, 63, 64, 65, 66, 67, 68, 69, 70, 71, 72, 73, 74, 75, 76

	Grazing yaks (LD)<br>NO

Positive samples in Dangxiong County	
Parasite	Sample lD
Cryptosporidium spp.	3, 4, 5, 8, 10, 11, 12, 15, 16, 17, 19, 21, 22, 23, 25, 26, 27, 31, 32, 33, 34, 35, 36, 37, 38, 39, 40, 41, 42, 43, 45, 46, 47, 49, 52, 53, 56, 57, 59, 60, 61, 63, 65
Giardia intestinalis	4, 7, 14, 18, 22, 24, 36, 42, 54
E. bieneusi	3, 4, 5, 7, 8, 9, 10, 11, 12, 13, 14, 17, 18, 19, 20, 21, 22, 24, 26, 27, 29, 30, 31, 32, 34, 35, 36, 37, 38, 40, 41, 42, 43, 44, 45, 46, 47, 48, 49, 52, 53, 54, 57, 60, 61, 62, 63, 66
Blastocystis spp.	2, 3, 4, 5, 6, 7, 8, 9, 10, 11, 12, 13, 14, 15, 16, 17, 18, 19, 20, 21, 22, 24, 25, 26, 27, 29, 30, 31, 32, 33, 34, 35, 36, 37, 38, 40, 41, 42, 43, 44, 45, 46, 47, 48, 49, 52, 53, 54, 56, 57, 58, 60, 61, 62, 63, 64, 65, 66

Positive samples in Nimu County	
Parasite	Sample lD
Cryptosporidium spp.	Captive yaks（N）<br>2, 3, 4, 5, 6, 8, 10, 11, 12, 13, 14, 15, 16, 19, 20, 21, 22, 24, 25, 26, 28, 29, 30, 31, 33, 34, 36, 38, 39, 41, 44, 45, 46, 47, 48, 56, 57, 60, 61, 62, 71, 72
	Grazing yaks (NF)<br>1, 2, 3, 5, 7, 8, 10, 11, 12, 15, 18, 20, 23, 24, 25, 26, 28, 30, 35, 36, 37, 39, 40, 41, 42, 43, 45, 46, 47, 48, 50, 52, 53, 56, 57, 59, 62, 66, 69, 70, 71, 72
Giardia intestinalis	Captive yaks（N）<br>1, 2, 3, 4, 6, 7, 8, 9, 10, 12, 13, 17, 19, 20, 21, 22, 23, 24, 30, 31, 32, 33, 35, 36, 40, 42, 43, 45, 49, 52, 58, 59, 60, 63, 64, 65, 67, 68, 69, 72, 74, 76, 78
	Grazing yaks (NF)<br>53
E. bieneusi	Captive yaks（N）<br>5, 6, 7, 8, 10, 11, 18, 19, 20, 21, 22, 23, 24, 25, 26, 28, 30, 31, 32, 41, 42, 44, 45, 53, 54, 55, 58, 60, 62, 65, 67, 69, 71, 72, 73, 75, 77, 78
	Grazing yaks (NF)<br>1, 3, 4, 5, 6, 10, 11, 12, 13, 14, 15, 16, 17, 18, 19, 20, 21, 23, 25, 26, 27, 28, 29, 30, 31, 32, 33, 35, 36, 37, 38, 39, 40, 42, 43, 44, 45, 46, 47, 48, 49, 50, 53, 55, 63, 64, 65, 70, 71
Blastocystis spp.	Captive yaks（N）<br>1, 2, 3, 4, 5, 6, 7, 8, 9, 10, 11, 12, 13, 14, 15, 16, 17, 18, 19, 20, 21, 22, 23, 24, 25, 26, 27, 28, 29, 30, 31, 32, 33, 34, 35, 36, 37, 38, 39, 40, 41, 42, 43, 44, 45, 46, 47, 48, 49, 50, 51, 52, 53, 54, 55, 56, 57, 58, 59, 60, 61, 62, 64, 65, 66, 67, 68, 69, 70, 71, 72, 73, 74, 75, 76, 77, 78
	Grazing yaks (NF)<br>1, 2, 3, 5, 6, 7, 8, 11, 13, 15, 18, 21, 24, 26, 27, 28, 29, 30, 31, 39, 40, 41, 42, 45, 46, 48, 49, 50, 52, 53, 54, 55, 56, 57, 59, 61, 62, 63, 64, 65, 67, 68, 72

Multiple parasitic infections

Positive samples in Linzhou County	
Parasite	Sample lD
Cryptosporidium spp. + *G. intestinalis*	Yak calves（LA）<br>No
	Adult yaks（LB）<br>No
	1–2-year-old yaks (LC)<br>2, 9, 14, 16, 18, 20, 21, 22, 28, 31, 32, 34, 35, 37, 39, 43, 44, 47, 51, 62
	Grazing yaks (LD)<br>No
Cryptosporidium spp. + *E. bieneusi*	Yak calves（LA）<br>7, 10, 13, 16, 20, 21, 30, 34
	Adult yaks（LB）<br>28, 30
	1–2-year-old yaks (LC)<br>2, 3, 8, 10, 12, 13, 14, 15, 16, 17, 18, 20, 21, 22, 24, 29, 32, 34, 38, 39, 40, 42, 43, 44, 46, 47, 51, 58, 60
	Grazing yaks (LD)<br>No
Cryptosporidium spp. + *Blastocystis* spp.	Yak calves（LA）<br>1, 2, 4, 6, 7, 10, 11, 13, 15, 16, 17, 18, 20, 22, 23, 24, 30, 31, 32, 34, 38, 40
	Adult yaks（LB）<br>2, 4, 8, 11, 12, 16, 22, 26, 27, 28, 30
	1–2-year-old yaks (LC)<br>1, 2, 3, 6, 8, 9, 10, 12, 13, 14, 15, 16, 17, 18, 20, 21, 22, 23, 24, 28, 29, 31, 32, 34, 35, 37, 38, 39, 40, 42, 43, 44, 46, 47, 49, 51, 54, 58, 60, 62, 64, 66, 67
	Grazing yaks (LD)<br>No
G. intestinalis + *E. bieneusi*	Yak calves（LA）<br>No
	Adult yaks（LB）<br>No
	1–2-year-old yaks (LC)<br>2, 11, 14, 16, 18, 19, 20, 21, 22, 25, 27, 32, 34, 39, 43, 44, 47, 48, 50, 51, 52, 53, 55, 56, 65
	Grazing yaks (LD)<br>5
G. intestinalis + *Blastocystis* spp.	Yak calves（LA）<br>No
	Adult yaks（LB）<br>No
	1–2-year-old yaks (LC)

	2, 9, 11, 14, 16, 18, 19, 20, 21, 22, 25, 28, 31, 32, 33, 34, 35, 37, 39, 41, 43, 44, 45, 47, 48, 50, 51, 52, 53, 55, 56, 59, 61, 62, 65
	Grazing yaks (LD)<br>No
E. bieneusi + *Blastocystis* spp.	Yak calves（LA）<br>7, 10, 13, 16, 20, 21, 30, 34, 36
	Adult yaks（LB）<br>6, 15, 21, 24, 25, 28, 29, 30, 37, 38
	1–2-year-old yaks (LC)<br>2, 3, 5, 7, 8, 10, 11, 12, 13, 14, 15, 16, 17, 18, 19, 20, 21, 22, 24, 25, 27, 29, 30, 32, 34, 36, 38, 39, 40, 42, 43, 44, 46, 47, 48, 50, 51, 52, 53, 55, 56, 57, 58, 60, 63, 65, 68, 69, 71, 72, 73
	Grazing yaks (LD)<br>No
Cryptosporidium spp. + *G. intestinalis* + *E. bieneusi*	Yak calves（LA）<br>No
	Adult yaks（LB）<br>No
	1–2-year-old yaks (LC)<br>2, 14, 16, 18, 20, 22, 32, 34, 39, 43, 44, 47, 51
	Grazing yaks (LD)<br>No
Cryptosporidium spp. + *G. intestinalis* + *Blastocystis* spp.	Yak calves（LA）<br>No
	Adult yaks（LB）<br>No
	1–2-year-old yaks (LC)<br>2, 9, 14, 16, 18, 20, 21, 22, 28, 31, 32, 34, 35, 37, 39, 43, 44, 47, 51, 62
	Grazing yaks (LD)<br>No
Cryptosporidium spp. + *E. bieneusi* + *Blastocystis* spp.	Yak calves（LA）<br>7, 10, 13, 16, 20, 21, 30, 34
	Adult yaks（LB）<br>28, 30
	1–2-year-old yaks (LC)<br>2, 3, 8, 10, 12, 13, 14, 15, 16, 17, 18, 20, 21, 22, 24, 29, 32, 34, 38, 39, 40, 42, 43, 44, 46, 47, 51, 58, 60
	Grazing yaks (LD)<br>No
G. intestinalis + *E. bieneusi* + *Blastocystis* spp.	Yak calves（LA）<br>No
	Adult yaks（LB）<br>No
	1–2-year-old yaks (LC)

	2, 11, 14, 16, 18, 19, 20, 21, 22, 25, 32, 34, 39, 43, 44, 47, 48, 50, 51, 52, 53, 55, 56, 65
	Grazing yaks (LD)<br>No
Cryptosporidium spp. + *G. intestinalis* + *E. bieneusi* + *Blastocystis* spp.	Yak calves（LA）<br>No
	Adult yaks（LB）<br>No
	1–2-year-old yaks (LC)<br>2, 14, 16, 18, 20, 22, 32, 34, 39, 43, 44, 47, 51
	Grazing yaks (LD)<br>No

Positive samples in Dangxiong County	
Parasite	Sample lD
Cryptosporidium spp. + *G. intestinalis*	4, 22, 36, 42
Cryptosporidium spp. + *E. bieneusi*	3, 4, 5, 8, 10, 11, 12, 17, 19, 21, 22, 26, 27, 31, 32, 34, 35, 36, 37, 38, 39, 40, 41, 42, 43, 45, 46, 47, 49, 52, 53, 57, 60, 61, 63
Cryptosporidium spp. + *Blastocystis* spp.	3, 4, 5, 8, 10, 11, 12, 15, 16, 17, 19, 21, 22, 25, 26, 27, 31, 32, 33, 34, 35, 36, 37, 38, 40, 41, 42, 43, 45, 46, 47, 49, 52, 53, 56, 57, 60, 61, 63, 65
G. intestinalis + *E. bieneusi*	4, 7, 14, 18, 22, 24, 36, 42, 54
G. intestinalis + *Blastocystis* spp.	4, 7, 14, 18, 22, 24, 36, 42, 54
E. bieneusi + *Blastocystis* spp.	3, 4, 5, 7, 8, 9, 10, 11, 12, 13, 14, 17, 18, 19, 20, 21, 22, 24, 26, 27, 29, 30, 31, 32, 34, 35, 36, 37, 38, 40, 41, 42, 43, 44, 45, 46, 47, 48, 49, 52, 53, 54, 57, 60, 61, 62, 63, 66
Cryptosporidium spp. + *G. intestinalis* + *E. bieneusi*	4, 22, 36, 42
Cryptosporidium spp. + *G. intestinalis* + *Blastocystis* spp.	4, 22, 36, 42
Cryptosporidium spp. + *E. bieneusi* + *Blastocystis* spp.	3, 4, 5, 8, 10, 11, 12, 17, 19, 21, 22, 26, 27, 31, 32, 34, 35, 36, 37, 38, 40, 41, 42, 43, 45, 46, 47, 49, 52, 53, 57, 60, 61, 63
G. intestinalis + *E. bieneusi* + *Blastocystis* spp.	4, 7, 14, 18, 22, 24, 36, 42, 54
Cryptosporidium spp. + *G. intestinalis* + *E. bieneusi* + *Blastocystis* spp.	4, 22, 36, 42

Positive samples in Nimu County	
Parasite	Sample ID
Cryptosporidium spp. + *G. intestinalis*	Captive yaks（N）<br>2, 3, 4, 6, 8, 10, 12, 13, 19, 20, 21, 22, 24, 30, 31, 33, 36, 44, 45, 59, 72
	Grazing yaks (NF)<br>53
Cryptosporidium spp. + *E. bieneusi*	Captive yaks（N）<br>5, 6, 8, 10, 11, 19, 20, 21, 22, 24, 25, 26, 28, 30, 31, 41, 44, 45, 60, 62, 71, 72
	Grazing yaks (NF)<br>1, 3, 5, 10, 11, 12, 15, 18, 20, 23, 25, 26, 28, 30, 35, 36, 37, 39, 40, 42, 43, 45, 46, 47, 48, 50, 53, 70, 71
Cryptosporidium spp. + *Blastocystis* spp.	Captive yaks（N）<br>2, 3, 4, 5, 6, 8, 10, 11, 12, 13, 14, 15, 16, 19, 20, 21, 22, 24, 25, 26, 28, 29, 30, 31, 33, 34, 36, 38, 39, 41, 44, 45, 46, 47, 48, 56, 57, 60, 61, 62, 71, 72
	Grazing yaks (NF)<br>1, 2, 3, 5, 7, 8, 11, 15, 18, 24, 26, 28, 30, 39, 40, 41, 42, 45, 46, 48, 50, 52, 53, 56, 57, 59, 62, 66, 72
G. intestinalis + *E. bieneusi*	Captive yaks（N）<br>6, 7, 8, 10, 19, 20, 21, 22, 23, 24, 30, 31, 32, 42, 44, 45, 60, 67, 72, 78
	Grazing yaks (NF)<br>53
G. intestinalis + *Blastocystis* spp.	Captive yaks（N）<br>1, 2, 3, 4, 6, 7, 8, 9, 10, 12, 13, 17, 19, 20, 21, 22, 23, 24, 30, 31, 32, 33, 35, 36, 40, 42, 43, 45, 49, 52, 58, 59, 60, 63, 64, 65, 67, 68, 69, 72, 74, 76, 78
	Grazing yaks (NF)<br>53
E. bieneusi + *Blastocystis* spp.	Captive yaks（N）<br>5, 6, 7, 8, 10, 11, 18, 19, 20, 21, 22, 23, 24, 25, 26, 28, 30, 31, 32, 41, 42, 44, 45, 53, 54, 55, 58, 60, 62, 65, 67, 69, 71, 72, 73, 75, 77, 78
	Grazing yaks (NF)<br>1, 3, 5, 6, 11, 13, 15, 18, 21, 26, 27, 28, 29, 30, 31, 39, 40, 42, 45, 46, 48, 49, 50, 53, 55, 63, 64, 65
Cryptosporidium spp. + *G. intestinalis* + *E. bieneusi*	Captive yaks（N）<br>6, 8, 10, 19, 20, 21, 22, 24, 30, 31, 45, 60, 72
	Grazing yaks (NF)<br>53
Cryptosporidium spp. + *G. intestinalis* + *Blastocystis* spp.	Captive yaks（N）<br>2, 3, 4, 6, 8, 10, 12, 13, 19, 20, 21, 22, 24, 30, 31, 33, 36, 44, 45, 59, 72
	Grazing yaks (NF)<br>53
Cryptosporidium spp. + *E. bieneusi* + *Blastocystis* spp.	Captive yaks（N）<br>5, 6, 8, 10, 11, 19, 20, 21, 22, 24, 25, 26, 28, 30, 31, 41, 44, 45, 60, 62, 71, 72
	Grazing yaks (NF)<br>1, 3, 5, 7, 8, 11, 15, 18, 26, 28, 30, 39, 40, 42, 45, 46, 48, 50, 53

G. intestinalis + *E. bieneusi* + *Blastocystis* spp.	Captive yaks（N）<br>6, 7, 8, 10, 19, 20, 21, 22, 23, 24, 30, 31, 32, 42, 44, 45, 60, 67, 72, 78
	Grazing yaks (NF)<br>53
Cryptosporidium spp. + *G. intestinalis* + *E. bieneusi* + *Blastocystis* spp.	Captive yaks（N）<br>6, 8, 10, 19, 20, 21, 22, 24, 30, 31, 45, 60, 72
	Grazing yaks (NF)<br>53
